# Cold Plasma as a Revolutionary Antimicrobial Modality: A Multifaceted Weapon Against Antibiotic Resistance

**DOI:** 10.3390/antibiotics14090930

**Published:** 2025-09-14

**Authors:** Yehia A.-G. Mahmoud, Nehal E. Elkaliny, Farah M. Elshikh, Yara Ashraf, Kamel Metwally, Galal Yahya, Sameha Sherif

**Affiliations:** 1 Botany and Microbiology Department, Faculty of Science, Tanta University, Tanta 31527, Egypt; 2 Applied and Analytical Microbiology Department, Faculty of Science, Ain Shams University, Ain Shams 11772, Egypt; 3 Department of Pharmaceutical Chemistry, Faculty of Pharmacy, University of Tabuk, Tabuk 71491, Saudi Arabia; 4 Department of Microbiology and Immunology, Faculty of Pharmacy, Zagazig University, Zagazig 44519, Egypt; 5 Botany and Microbiology Department, Faculty of Science, Zagazig University, Zagazig 44519, Egypt

**Keywords:** antibacterial, cold plasma, antibiotic resistance, reactive oxygen species, apoptosis

## Abstract

The rise of antibiotic resistance has transformed once-curable infections into urgent global health threats, leaving hospitals with outbreaks, patients with prolonged illnesses, and doctors with limited therapeutic options. The era of antibiotic resistance is no longer a distant concern; it is a pressing reality demanding innovative solutions. Among emerging alternatives, cold plasma a partially ionized state of matter enriched with reactive species offers a multi-targeted antimicrobial strategy. Unlike conventional antibiotics, cold plasma disrupts bacterial survival through diverse mechanisms, including membrane rupture, protein and nucleic acid damage, and oxidative stress that overwhelms microbial defenses. This review synthesizes current evidence on the mechanisms of cold plasma, the factors influencing its antimicrobial efficacy, and its applications across healthcare, food safety, and environmental protection. In addition, it highlights the synergistic potential of cold plasma when combined with antibiotics, nanomaterials, or bacteriophages to enhance effectiveness against resistant pathogens. While challenges remain regarding safety validation, standardization, and large-scale application, cold plasma represents a promising non-traditional approach to complement existing therapies. This review not only summarizes recent progress but also outlines future directions, emphasizing its potential role in combating antibiotic resistance.

## 1. Introduction

Antibiotics transformed healthcare in the 20th century by turning severe bacterial infections into manageable conditions. However, their extensive and often inappropriate use has contributed to the emergence of antibiotic-resistant bacteria, which the World Health Organization has identified as a major global health threat [1]. Increasing numbers of multidrug-resistant (MDR) and pan-resistant strains now compromise the effectiveness of conventional treatments, raising concerns for procedures such as surgery, chemotherapy, and organ transplantation [2].

This situation highlights the urgent need for alternative antimicrobial strategies. One of the approaches that has gained significant attention is cold plasma [3,4]. Plasma, the fourth state of matter, consists of a partially ionized gas containing electrons, ions, neutral atoms, ultraviolet (UV) photons, and reactive oxygen and nitrogen species (ROS and RNS). In contrast to thermal plasma, cold atmospheric plasma (CAP) operates at near-room temperature, which makes it suitable for biomedical applications without causing thermal damage to tissues [5].

The antimicrobial action of CAP is based on multiple mechanisms. Reactive species generated during plasma discharge damage bacterial membranes, leading to structural disruption [6]. ROS and RNS further penetrate cells and cause oxidative modifications of proteins, lipids, and nucleic acids [7,8]. In addition, CAP interferes with cellular metabolism and may induce apoptosis-like pathways in bacterial cells [9]. Since these effects are not directed at specific bacterial targets, the risk of resistance development is considered to be low [10]. CAP has therefore demonstrated efficacy against a wide range of bacteria, including multidrug-resistant species.

Research into CAP has extended beyond clinical applications and now covers diverse areas including sterilization, wound healing, dentistry, food preservation, agriculture, and environmental decontamination [11,12]. This review provides an updated overview of CAP in bacterial inactivation, with emphasis on its mechanisms of action, factors that influence antimicrobial efficacy, and applications across different sectors. We also discuss potential synergistic strategies with other antimicrobial approaches. The central guiding question is whether cold plasma can be developed as a practical antimicrobial alternative to antibiotics in both clinical and industrial contexts.

## 2. Mechanisms of Cold Plasma Action on Bacteria

Plasma is widely regarded as the fourth fundamental state of matter, following solids, liquids, and gases in the classical classification scheme [12,13]. It is typically observed as a luminous, spark-like glow due to its energetic nature [12]. The transition between states of matter is driven by the energy supplied to a substance. When energy is added to a solid, atoms gain kinetic energy, intermolecular bonds break, and the substance shifts to the liquid state. Continued energy input increases evaporation beyond condensation, producing a gas. With further excitation, gas atoms move faster and collide more forcefully, ejecting electrons and creating an ionized state known as plasma [13,14].

Plasma is composed of a dynamic mixture of fully or partially ionized gases, including free electrons, ions, neutral atoms and molecules, reactive species, and photons, all of which contribute to its unique chemical and physical reactivity [12,13]. The composition and behavior of plasma can vary significantly depending on both the type of feed gas used—such as nitrogen, helium, argon, or compressed air—and the configuration of the plasma generation system [15]. Indeed, the properties of the resulting plasma are heavily influenced by several reactor parameters, including the geometry of the chamber, the gas flow rate, and the frequency of the applied energy source [16]. These factors collectively determine the physicochemical characteristics of the plasma and, consequently, its interaction with biological or material surfaces.

Plasma can be broadly categorized into two main types: high-temperature plasma, commonly referred to as hot plasma, and non-thermal plasma, also known as cold plasma [17]. Thermal plasma is characterized by the presence of thermal equilibrium, where electrons, ions, and neutral particles all possess approximately the same temperature [17]. In contrast, non-thermal plasma exhibits a distinct lack of thermal equilibrium, as there exists a significant disparity in temperature between the various plasma components—particularly between electrons, which are highly energetic, and heavier species such as ions and gas molecules [17,18,19].

Cold plasma, also referred to as low-temperature plasma, is typically produced at or near room temperature and does not exceed 40 °C in most cases. It is generated by applying high-voltage electrical discharges, usually ranging from 10 to 20 kV, in combination with low electric currents not exceeding 1–2 A [18,20,21]. A variety of plasma generation systems have been developed to produce cold plasma under atmospheric conditions, including Atmospheric Pressure Glow Discharges (APGD), Atmospheric Pressure Plasma Jets (APPJ), Dielectric Barrier Discharges (DBD), Gliding Arc Discharges (GAD), and Pulsed Discharges (PD) [22]. Among these, the DBD and APPJ systems are the most widely employed due to their relatively simple configurations, ease of customization, and adaptability to various applications. These features make them particularly suitable for diverse industrial and biomedical uses, such as in the food sector, agriculture, and medical treatments [23].

Cold plasma is considered as a promising technology to inactivate microorganisms including those strains that are Multi drug resistant [24,25]. In a study conducted by Punam Talukdar et al., they found that cold plasma could inactivate *E. coli*, *S. aureus* and *C. albicans* as after treating these microorganisms with cold plasma, inhibition zones were observed which were at a level comparable to, or better than traditional antibiotics [25]. Also, in another study conducted by McKayla J. Nicol et al., the authors found that cold plasma is a potential alternative to traditional antibiotics especially for treating infections caused by multi drug resistant bacteria as bacteria, such as *E. coli* do not develop resistance to cold plasma unlike antibiotics as cold plasma remains effective even after multiple exposures [26].

### 2.1. Generation of Reactive Species

Cold plasma has been extensively demonstrated to possess broad-spectrum antimicrobial activity, exerting inhibitory effects against a wide range of microorganisms including bacteria, fungi, yeasts, viruses, and even bacterial spores [23,27]. Although the exact mechanisms underlying this antimicrobial activity remain not fully elucidated, it is hypothesized that several components—such as reactive oxygen species (ROS), reactive nitrogen species (RNS), hydrogen peroxide (H_2_O_2_), and UV radiation—play key roles in microbial inactivation [15,28].

Plasma-generated reactive species are generally categorized into two types: short-lived and long-lived species [29]. Both types contribute to the antibacterial action of plasma, although long-lived species retain their reactivity over extended periods, enhancing their stability and potential impact [7]. In contrast, short-lived species exhibit a significantly reduced lifespan, limiting their diffusion capacity in liquid environments. As a result, their ability to penetrate into aqueous solutions is restricted. Nevertheless, these species can still exert indirect antimicrobial effects through the formation of secondary reactive intermediates following their interaction with liquids [29].

Cold plasma is capable of generating a diverse array of reactive chemical species. Among these, the ROS include hydroxyl radicals (^•^OH), H_2_O_2_, singlet oxygen (^1^O_2_), superoxide anions (O_2_^−^), and ozone (O_3_). Meanwhile, the RNS comprise nitric oxide (NO), nitrogen dioxide (NO_2_), nitrous oxide (N_2_O), nitrogen trioxide (NO_3_), dinitrogen tetroxide (N_2_O_4_), and peroxynitrite (ONOO^−^) [17,30].

**Figure 1:**
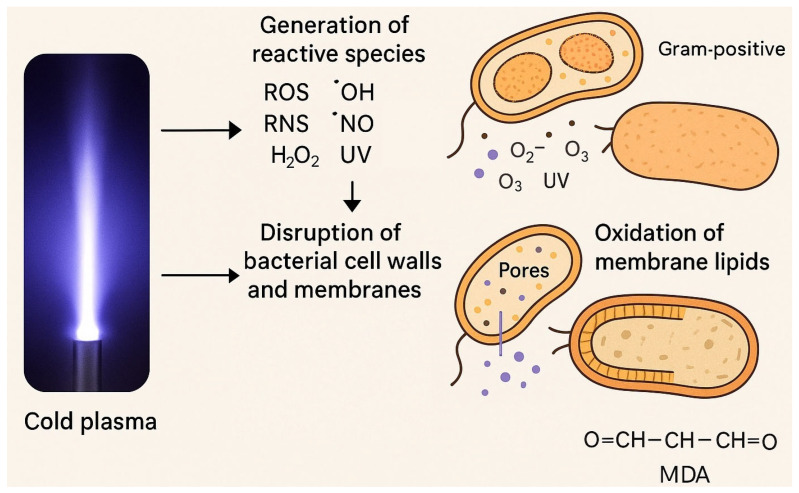
Antibacterial mechanisms of cold plasma, showing its effects on bacterial membranes, intracellular components, and metabolic pathways.

Some of these species arise from the interaction of plasma with gas-phase components—for example, the formation of hydroxyl radicals and nitric oxide—while others are generated through plasma–liquid interactions, resulting in compounds such as hydrogen peroxide, nitrites, and nitrates [17] (Figure 1).

### 2.2. Disruption of Bacterial Cell Walls and Membranes

Cold plasma inactivates microorganisms through two main mechanisms: physical disruption and biochemical damage. In the physical pathway, pores form in the bacterial cell membrane, causing leakage of intracellular contents and loss of integrity. In the biochemical pathway, reactive species attack key cellular components, including the cell wall, membrane lipids, proteins, and DNA [31,32,33].

Several parameters influence the efficacy of plasma-based microbial inactivation. These include the characteristics of the electrical discharge (such as voltage, frequency, and current), the mode of exposure (direct versus indirect), the duration of plasma treatment, the concentration of bacterial cells, and the specific bacterial species involved [7]. Notably, Anne Mai-Prochnow and colleagues reported that the susceptibility of bacterial cells to cold plasma is closely related to the structural thickness of their cell walls, with thinner walls being more easily compromised [15].

A number of studies have consistently shown that Gram-negative bacteria are generally more susceptible to cold plasma treatment than Gram-positive bacteria [15,34]. This differential sensitivity is attributed to variations in cell wall architecture. Gram-positive bacteria possess a thick peptidoglycan layer ranging from 20 to 80 nm, which enhances their resistance to both chemical and physical stressors. In contrast, Gram-negative bacteria have a much thinner peptidoglycan layer, typically between 2 and 3 nm, making them more vulnerable to plasma-induced damage [34,35].

Cold plasma also induces oxidative stress in bacterial cells by attacking the lipid bilayer of the membrane. During plasma exposure, reactive species oxidize membrane lipids, producing malondialdehyde (MDA), a highly reactive aldehyde. Unlike most reactive species, MDA is chemically stable and can penetrate the cytoplasm, where it interacts with proteins and nucleic acids, amplifying the antimicrobial effect of cold plasma [6] (Figure 1).

This antimicrobial efficacy of cold plasma has been further validated by several recent studies. In one study conducted in 2025, researchers demonstrated that cold plasma can effectively inactivate *Pseudomonas aeruginosa* through a combination of mechanisms including oxidation of the lipid membrane, disruption of intracellular ion balance, alteration of membrane permeability, and morphological deformation of the bacterial cells [30].

In another investigation by L. Han et al., high-voltage atmospheric cold plasma was applied to two bacterial strains: *Escherichia coli* NCTC 12900 (a non-toxigenic O157\:H7 Gram-negative strain) and *S. aureus* ATCC 25923 (a Gram-positive strain). Their findings revealed that ROS generated by cold plasma disrupted the structural integrity of *E*. *coli* by cleaving C–C, C–O, and C–N bonds within both the lipopolysaccharide layer and the peptidoglycan layer of the cell wall. Additionally, the lipid components of the bacterial envelope underwent peroxidation, ultimately leading to collapse of the cell envelope [33] (Figure 1).

Interestingly, *S. aureus* exhibited a different pattern of response. The study showed that ROS penetrated the Gram-positive cell wall and accumulated within the intracellular environment, causing damage primarily to internal components rather than the cell envelope. Notably, the intracellular ROS concentration in *S. aureus* was found to be higher than that in *E. coli*, which partially explains why the outer structure of Gram-positive bacteria is less affected compared to that of Gram-negative bacteria [33].

Further evidence of cold plasma’s mechanism of action was presented in a 2023 study focusing on *Bacillus cereus*, a Gram-positive bacterium. The inactivation process occurred in three sequential stages: initially, plasma exposure led to structural breakdown of the cell membrane and visible morphological alterations. Subsequently, ROS penetrated the cell, attacking vital macromolecules such as proteins and nucleic acids. This internal damage disrupted cellular homeostasis, ultimately resulting in cell death [31].

Consistent findings were also reported by Marlène Dezest et al., who studied the effect of helium-based cold plasma on *E. coli* CIP 53126. After a 10 min exposure followed by 2 h of storage, complete bacterial inactivation was observed. Cold plasma treatment resulted in pore formation within the membrane, leading to total membrane disruption. Moreover, morphological changes were evident, as some bacterial cells lost their characteristic rod-shaped (bacillus) form and transformed into a coccoid morphology [36]. Summary of the representative effects of CAP on different bacterial species is presented in Table 1.

### 2.3. Oxidative Stress and Intracellular Damage

As previously discussed, the antimicrobial effect of cold plasma is largely attributed to the generation of reactive species. A study conducted in 2019 investigating the impact of cold plasma on *Listeria monocytogenes* revealed that plasma exposure led to the intracellular accumulation of ROS, including H_2_O_2_ and O_2_^−^. These initial ROS triggered a cascade of redox reactions, resulting in the formation of large quantities of highly reactive, short-lived species such as ^•^OH. These radicals induced severe oxidative damage to internal cellular structures, leading to cell death [37].

The presence of ROS within bacterial cells leads to the development of oxidative stress, a condition characterized by the disruption of redox balance and the subsequent damage to essential biomolecules such as lipids, proteins, and nucleic acids [37,38]. Oxidative stress is defined as an imbalance between the production of oxidants and the capacity of the cell’s antioxidant defense systems to neutralize them [39]. It occurs when the generation or accumulation of ROS surpasses the cell’s detoxification capabilities, resulting in the loss of cellular homeostasis. Although all living organisms experience oxidative stress, the extent of damage and the response mechanisms vary across species and depend on the origin of the stress—whether endogenous, such as from aerobic respiration, or exogenous, such as exposure to antimicrobial agents or host immune responses [40,41].

**Figure 2:**
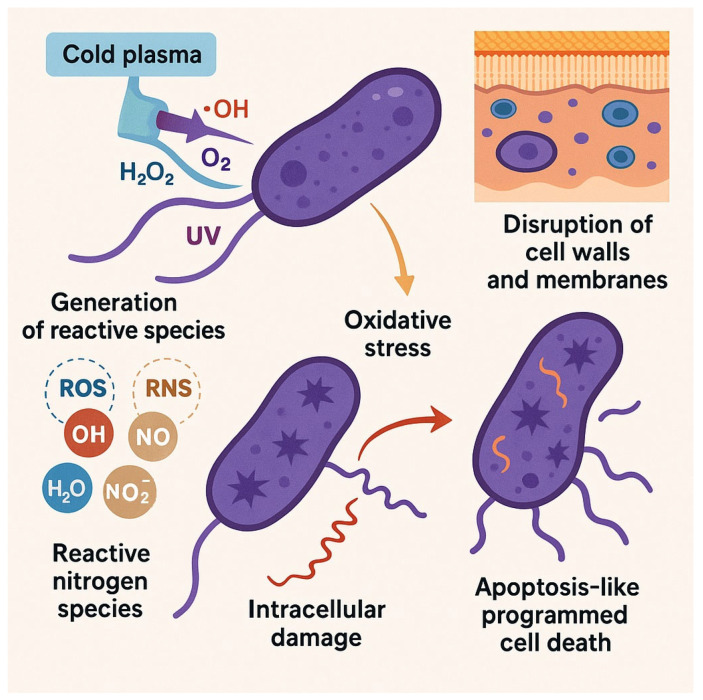
Oxidative Stress and Intracellular Damage Induced by Cold Plasma.

Interestingly, some conventional antimicrobial agents—including certain antibiotics—exert their effects through ROS generation. For example, Nitrofurantoin, used in urinary tract infections, and Polymyxin B, employed against Gram-negative infections, both mediate bacterial inactivation through ROS production. However, with the widespread and often inappropriate use of antibiotics, alternative antimicrobial agents have emerged that target bacterial redox systems, thereby enhancing ROS accumulation and oxidative damage. One such target is the thioredoxin reductase enzyme (TrxR), which plays a crucial role in maintaining intracellular redox homeostasis [42] (Figure 2).

Despite the harmful potential of ROS, bacterial cells possess intricate defense systems to mitigate low levels of oxidative stress. In some cases, sublethal concentrations of ROS may not be detrimental; instead, they can act as signaling molecules, modulating key bacterial processes such as quorum sensing, biofilm formation, and even programmed cell death [41]. This dual role of ROS—as both antimicrobial agents and intracellular messengers—highlights the complexity of oxidative stress and its significance in bacterial physiology.

To counteract oxidative stress, bacteria activate a range of transcriptional regulators that coordinate the expression of defense-related genes. Among the most studied regulators are SoxRS, OxyR, and PerR, which are responsible for modulating the bacterial response to elevated levels of ROS [41,43]. The genes controlled by these regulators are categorized based on their expression dynamics in response to oxidative stress. These categories include: down regulated genes, pulsatile upregulated genes—which are transiently expressed in a subset of cells to confer short-term protection to neighboring populations—and gradually upregulated genes, which provide sustained cellular defense against oxidative damage [41,43].

A central strategy employed by bacteria to survive oxidative stress involves the production of antioxidant enzymes that neutralize ROS into non-toxic compounds. One key enzyme is catalase, which plays a critical role in decomposing H_2_O_2_ into water and molecular oxygen. The reaction proceeds through a two-step catalytic mechanism, as extensively described by Fang Yuan et al. [43]. Another important group of enzymes are the superoxide dismutases (SODs), which are metalloenzymes that convert O_2_^−^ into H_2_O_2_ and oxygen. The H_2_O_2_ generated is subsequently degraded by catalase or peroxidase enzymes [44] (Figure 2).

This diagram outlines the intracellular effects of ROS generated by cold plasma treatment. It highlights ROS accumulation, redox imbalance, and resulting oxidative damage to lipids, proteins, and nucleic acids. The figure also depicts the bacterial antioxidant defense mechanisms—both enzymatic (e.g., catalase, superoxide dismutase) and non-enzymatic (e.g., glutathione)—and how their capacity influences species-specific responses. Additionally, it shows the modulation of gene expression through key transcriptional regulators (SoxRS, OxyR, and PerR).

Antioxidant systems in bacteria are broadly divided into enzymatic and non-enzymatic defenses. The non-enzymatic antioxidants include small molecules such as ascorbic acid, glutathione, and α-tocopherol [40]. Among them, reduced glutathione (GSH) is particularly effective, as it can directly scavenge free radicals, preventing them from interacting destructively with intracellular components.

In a study conducted in 2022, researchers investigated the impact of oxidative stress on the production levels of reduced glutathione, MDA, and enterobactin siderophore across different bacterial species exposed to hydrogen peroxide. The results showed that *S. aureus* was the least affected, displaying minimal changes in both GSH and MDA levels upon H_2_O_2_ exposure. In contrast, *E. coli* ATCC 25922 exhibited a substantial increase in the levels of both reduced glutathione and malondialdehyde, suggesting a strong oxidative response. Conversely, *Klebsiella pneumoniae* ATCC 700603 showed a marked decrease in these molecules following hydrogen peroxide exposure [40]. The authors attributed these differences to structural variations in bacterial cell envelopes. Specifically, the thick peptidoglycan layer in *S. aureus* is thought to limit the diffusion of H_2_O_2_ into the cytoplasm, thereby offering enhanced protection against oxidative stress [40].

### 2.4. Effects of Cold Plasma on Bacterial DNA and Proteins

As previously discussed, the impact of cold plasma on bacterial cells varies significantly depending on the bacterial type. In a study by L. Han et al., it was demonstrated that in *E. coli*—a Gram-negative bacterium—the primary site of plasma-induced damage is the cell wall. This damage leads to leakage of intracellular contents; however, only minor effects on the genomic DNA were observed. In contrast, in *S. aureus*, a Gram-positive bacterium, cold plasma caused substantial intracellular damage, particularly to the DNA, without inducing membrane leakage. This differential response is attributed to the higher intracellular accumulation of ROS in Gram-positive cells compared to Gram-negative ones [33].

Further insights were provided by H. Lu et al., who evaluated the influence of high-voltage cold plasma on the genomic DNA of *E. coli* and *Listeria monocytogenes* (a Gram-positive species). The study highlighted that DNA damage induced by cold plasma is influenced by both the bacterial type and the exposure duration. When bacterial cells were exposed to cold plasma for 5 s, the treatment primarily affected the bacterial membrane, leading to leakage of cellular contents without notable damage to genomic DNA. The authors suggested that this short exposure time was insufficient for ROS to penetrate and damage DNA, especially since both *E. coli* and *L. monocytogenes* possess enzymatic defense systems—such as alkyl hydroperoxide reductase, superoxide dismutase, and catalase—that can neutralize ROS [45].

However, when the exposure duration was increased to 30 s, significant genomic DNA damage was observed in *L. monocytogenes*, while *E. coli* exhibited only minimal DNA damage. This suggests that the multilayered membrane of Gram-negative bacteria may offer enhanced protection, and that longer exposure times are required to induce nucleic acid damage in such species [45].

A study by Bethany L. Patenall et al. further emphasized the role of exposure time in DNA damage. When *E. coli* was subjected to CAP for less than 5 min, only a limited number of mutations were detected. In contrast, longer exposure durations led to a substantial increase in DNA mutations. The study concluded that ROS and RNS were the primary contributors to this genotoxicity, rather than UV radiation, although some UV is generated during plasma treatment [46]. This conclusion contrasts with the findings of Nathalia M. Coutinho et al., who proposed that cold plasma inhibits bacterial replication through thymine dimer formation and DNA strand breaks, caused synergistically by both UV radiation and free radicals [47].

To investigate the specific contribution of UV damage, Patenall et al. utilized an *E. coli* mutant lacking the *uvrA* gene, which is involved in the recognition and repair of UV-induced DNA lesions [46,48]. Following CAP treatment, the mutant strain exhibited only a 5–10-fold increase in mutation frequency compared to the wild-type strain. Based on these results, the authors concluded that UV radiation is not the predominant factor contributing to DNA damage under their experimental conditions [46].

### 2.5. Induction of Apoptosis-like Cell Death in Bacteria

As previously described, cold plasma is a dynamic mixture of charged particles—mainly ions and electrons—that generate an electrostatic field able to disrupt chemical bonds in the bacterial cell wall. This disruption causes structural damage, including pore formation and surface erosion, which facilitates the entry of toxic reactive species into the cytoplasm [49]. Once inside the cell, ROS initiate oxidative stress by attacking membrane lipids, denaturing proteins, and damaging nucleic acids such as DNA. These molecular-level alterations result in profound morphological changes and loss of cellular function. Eventually, the cumulative damage overwhelms the bacterial repair systems and leads to irreversible cell death [49].

In addition to its bactericidal effects, cold plasma has also demonstrated the ability to interfere with bacterial gene expression, particularly genes related to pathogenicity. A study conducted in 2021 highlighted cold plasma as a promising alternative to antibiotics, noting its ability to inhibit microbial growth without contributing to antibiotic resistance. The researchers observed that exposure of *P. aeruginosa* to cold plasma led to a significant downregulation of virulence-associated genes. Specifically, the expression of the alp gene was reduced 1.5-fold following treatment, indicating that cold plasma may attenuate bacterial virulence in addition to reducing viability [50]. This dual action—disruption of cell integrity and suppression of pathogenic mechanisms—positions cold plasma as a powerful antimicrobial strategy with broad therapeutic potential (Figure 2).

### 2.6. Clarification of Certainty in Mechanisms

The antimicrobial action of cold plasma is supported by both well-established and emerging mechanisms. Among the well-established processes are the generation of ROS and RNS, oxidative damage to bacterial membranes, disruption of cell wall integrity, leakage of intracellular contents, and subsequent impairment of vital biomolecules including proteins and nucleic acids [31,33,51]. These effects have been consistently demonstrated across multiple bacterial species and study designs, indicating a high level of certainty. In contrast, other proposed mechanisms remain under investigation. For example, apoptosis-like cell death in bacteria and plasma-mediated modulation of bacterial gene expression, such as the downregulation of virulence-associated genes, have been reported [49,50], but the molecular pathways underlying these effects are not yet fully elucidated and require further validation. Distinguishing between these established and exploratory mechanisms is important to accurately frame the current state of knowledge and identify priorities for future research.

## 3. Factors Affecting Bacterial Inactivation by Cold Plasma

**Figure 3:**
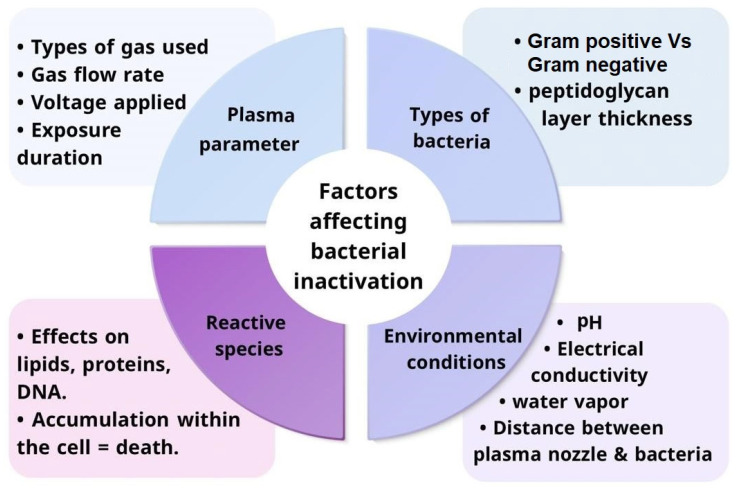
Factors Affecting Bacterial Inactivation by Cold Plasma.

The application of the cold plasma approach for bacterial inactivation depends on many factors that have either a positive or negative impact on bactericidal. The factors are represented in two categories: primary factors and secondary factors according to their degree of influence. The factors are represented in four categories: plasma parameters, type of bacteria, environmental conditions, and reactive species generated. These factors must be adjusted to achieve optimal efficiency with bacterial elimination. Figure 3 Summaries factors that affects bacterial inactivation using cold plasma.

### 3.1. Primary Factors

#### 3.1.1. Plasma Parameters and Their Influence on Bacterial Inactivation

Plasma is a neutral gas that is either partially or fully ionized via the application of electrical energy, consisting of free electrons, ions, atoms, neutral molecules, and photons in a metastable or excited state. Plasma is classified into two categories according to electron temperature: high-temperature plasma and low-temperature plasma, which has two subunits based on thermodynamic equilibrium state, thermal plasma and non-thermal plasma (cold). Thermal plasma is essential for pyrolytic processes and other high-temperature applications in metallurgy, high-temperature chemistry, thermal spraying, and other fields. However, cold plasma plays an important role in some applications where it needs to interact with solid substrates without damaging them, as in the case of bacterial inactivation [52]. Cold plasma exists in a local thermodynamic non-equilibrium state, and its electron temperature is higher than 104 K [53]. The selection of gas utilized in cold plasma treatment influences the outcomes, such as ionization efficiency, UV emission intensity, and the creation of reactive species. Noble gases such as helium and argon are mostly used due to their UV emission spectra and excellent thermal conductivity [54]. The ionization potential of noble gases is low, resulting in lower electron temperatures that allow the acceleration of chemical reactions in them for ROS generation [55,56]. Additionally, excitation and ionization reactions happen at a high rate in the case of the presence of noble gases [57]. The presence of air with gas in the mixture results in reducing the bactericidal power of cold plasma. This deficiency reduction is due to the electronegative nature of O_2_, which causes electron attachment loss to prevail over electron impact dissociation beyond an optimal threshold, resulting in a reduction in atomic oxygen radicals [58]. Gas rate flow is a vital parameter in cold plasma treatment that accelerates the speed of species to reach the target surface [55]. When the flow rate of helium (He) gas was raised from 10 L per minute to 14 L per minute, the survival count fell from 5 × 10^8^ CFU/mL to 6 × 10^2^ CFU/mL for *E. coli* and from 5 × 10^8^ CFU/mL to 7 × 10^3^ CFU/mL for *S. aureus*. Since the flow rate is increasing, more radical species are produced, which explains the drop in bacterial cells. The duration of cold plasma exposure also influences bacterial inactivation. Higher exposure to cold plasma has, a more significant bacterial-killing impact [51]. The power of voltage in a cold plasma device when increased from 15 kV to 21 kV raises the bactericidal rate [59].

#### 3.1.2. Role of Reactive Species in Bacterial Inactivation

Two reactive species are formed during the energy transfer and gas excitation: nitrogen reactive species as NO radicals, atomic N, and oxygen reactive species as OH radicals, atomic O, and O_3_. Cold plasma treatment is greatly influenced by these reactive species that are responsible for bacterial cell wall damage. Addition of water in minute amounts with noble gases will achieve better production of reactive species [54]. Higher energy input, including voltage and frequency, increases the amount of reactive species formation that affects the inactivation rate [46]. Bacterial inactivation can be accelerated via numerous reactive species that work either independently or synergistically [60]. Singlet oxygen is the most important reactive species in the bacterial inactivated process because it is easy to activate and has a long lifespan. Because of the unique electronic structure and excess energy of singlet oxygen make it highly reactive against macromolecules with high electron-contained double bonds, such as proteins, DNA, and lipid acids [59]. These findings are summarized in Table 2, which outlines the major reactive species generated during CAP treatment, their flow rate effects, and the resulting bacterial responses.

### 3.2. Secondary Factors

#### 3.2.1. Bacterial Types and Their Response to Cold Plasma

**Figure 4:**
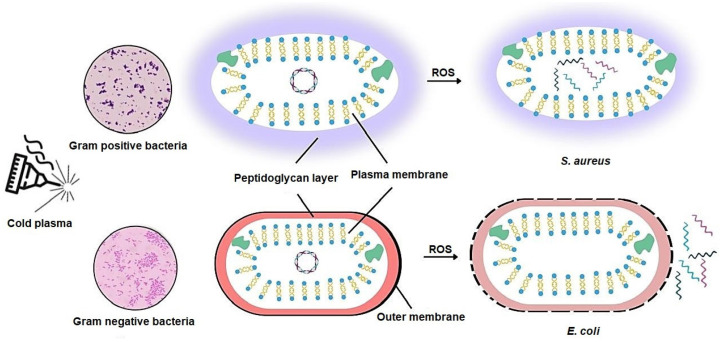
Effect of Cold Plasma on Gram-positive and Gram-negative bacteria.

Bacterial response to the cold plasma depends on the bacterial cell wall structure. The more peptidoglycan layers, the less susceptible to cold plasma treatment. Gram-positive bacteria have a 20–80 nm peptidoglycan layer, and Gram-negative bacteria have a less than 10 nm peptidoglycan layer. Cold plasma works better against Gram-negative bacteria because their peptidoglycan layer is smaller than that of Gram-positive bacteria. For instance, when exposed to cold plasma, the Gram-negative bacterium *E. coli*, which has a thin peptidoglycan coating, develops cell leakage. This happens as a result of irreversible oxidation of the peptidoglycan layer and damage to the lipopolysaccharides in the outer cell membrane [50]. The thin layer of Gram-negative cell wall structure makes the Gram-negative bacteria more susceptible to ROS damage and UV radiation. As a result, the Gram-negative cell contents, like protein, lipid, and low level DNA, are released [61,62]. The large peptidoglycan layer in Gram-positive prevents the flowing of cold plasma reactive species via its cellular membranes. Despite that, some studies have shown that controlling the exposure duration to cold plasma can inactivate both Gram-positive and Gram-negative [54] (Figure 4). In Gram-positive bacteria like *S. aureus*, cold plasma causes intracellular damage to proteins and genetic material that results in bactericidal. Essential oils are secondary metabolites produced by plants that, when extracted and combined with cold plasma, show more effectiveness against Gram-positive bacteria. The preliminary information indicates that combining cold plasma with essential oils amplifies its efficacy against Gram-positive bacteria, although this synergy necessitates additional validation [54] (Figure 4).

#### 3.2.2. Impact of Environmental Conditions on Cold Plasma Efficiency

There are many other factors affecting bacterial inactivation, like electroporation, UV radiation, charged particles, or short-lived species being decomposed in the absence of target cells. Physiochemical properties such as pH and electrical conductivity must be adjusted for optimal operation. In addition, the source frequency, voltage, and power employed can affect discharge properties and chemical features of cold plasma and bacterial inactivation power. Acidic pH helps form reactive species and increases electrical conductivity [63]. PH is considered a vital factor in bacterial inactivation because an acidic pH lower than 4.7 facilitates the penetration of reactive species into the bacterial cell wall and causes denaturation of protein structures and cell leakage [51,61]. At low pH, the superoxide anion radical (O_2_^•−^) undergoes protonation that results in hydroperoxy radical (HOO^•^) formation. As a result of the uncharged HOO^•^, it can diffuse into the bacterial cell by traversing the hydrophobic cell membrane and causing chemical modification to proteins, resulting in protein denaturation and bacterial damage [64]. Some studies have demonstrated that shortening the duration of the cold plasma treatment to less than 4 min can inactivate bacteria with no toxic effect on humans due to reducing pH or heat, and does not cause inflammation or DNA damage [61]. Water vapor could induce H_2_O_2_ formation that participates in bacterial cell wall oxidation. The distance between the cold plasma nozzle and the target bacteria is a vital factor in the process; due to the higher distance, there is a lower potential for inactivated bacteria [51]. Increasing power supply is important in bacterial inactivation ability [58].

## 4. Applications of Cold Plasma in Bacterial Treatment

### 4.1. Medical and Healthcare Applications

As previously discussed, cold plasma exhibits strong antimicrobial properties, enabling it to inhibit the growth of a wide spectrum of microorganisms. This makes it highly suitable for use in sterilization and microbial disinfection. Beyond its antimicrobial potential, cold plasma has been explored for various biomedical and clinical applications, such as wound healing, treatment of skin diseases, suppression of angiogenesis, cancer therapy, management of dental diseases, immunotherapy, and even as a blood clotting agent [21,65,66,67].

**Figure 5:**
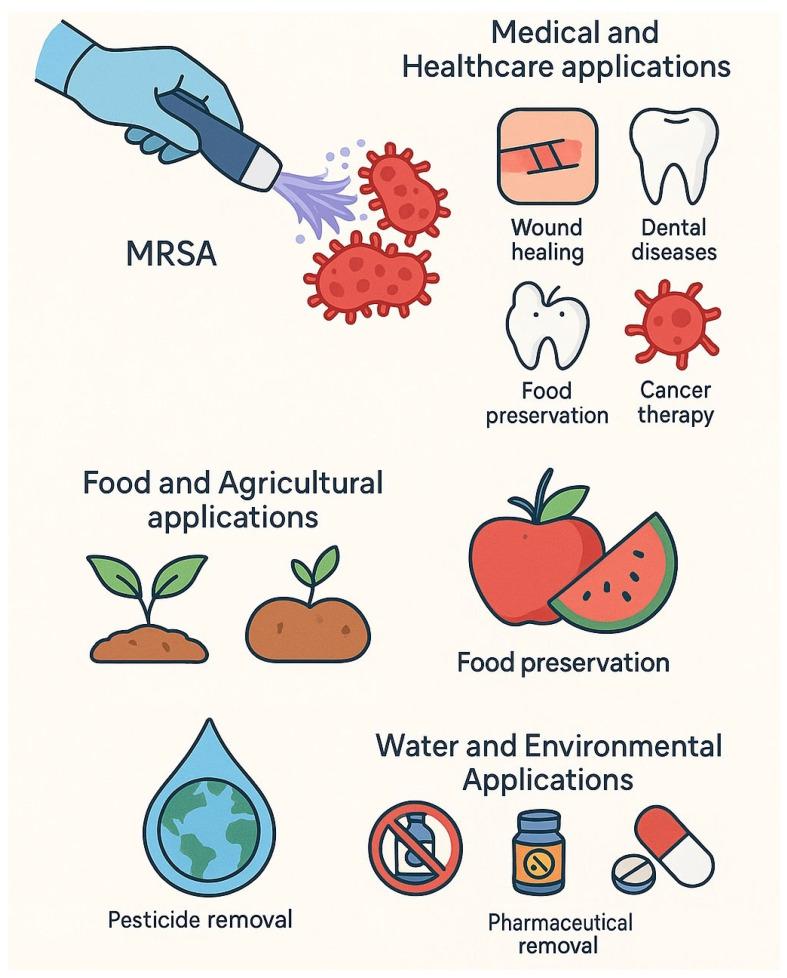
Antibacterial Potential of cold plasma in Healthcare, Food, Agricultural, and Environmental Applications.

In the field of sterilization, cold plasma has gained considerable attention as a non-thermal, non-damaging alternative to traditional methods like autoclaving. A study conducted in 2021 investigated the efficacy of cold plasma in sterilizing surgical implants. In this study, a stainless-steel loop contaminated with *Pasteurella multocida* was sterilized using cold plasma and subsequently implanted subcutaneously in rabbits. The results demonstrated complete inactivation of the bacterial pathogen without inducing any visible tissue damage or erythema in the epidermis, dermis, subcutis, or muscle layers. This highlighted cold plasma’s safety and efficiency as a sterilization method. However, the authors emphasized the need for further studies to assess its effectiveness against a broader range of bacterial species [67] (Figure 5). This suggests that CAP can serve as a safe and effective sterilization approach for surgical materials, although validation against a wider spectrum of pathogens is still required.

Cold plasma also shows promise in environmental decontamination, particularly in hospital settings, due to its safety profile and eco-friendly nature. A 2023 study by M. Lunder et al. Demonstrated that cold plasma could effectively eradicate both *Staphylococcus aureus* and multidrug-resistant MRSA biofilms. The mechanism of action involved membrane disruption, DNA damage, and ROS-induced oxidative stress. Notably, no resistance to cold plasma treatment was observed, in contrast to the increasing resistance observed with conventional antibiotics. Despite these encouraging results, the authors recommended further investigations into the use of cold plasma for eliminating polymicrobial biofilms in real-world hospital environments [10]. These findings strengthen the case for CAP as a hospital disinfection tool, particularly against antibiotic-resistant organisms; however, its performance against complex polymicrobial biofilms in real hospital environments remains to be determined.

In dentistry, cold plasma has been evaluated as a potential alternative to peroxide-based bleaching agents. A 2024 in vitro study by Seoul-Hee Nam et al. assessed the whitening effects of distilled water treated with cold plasma. The treated water exhibited a strong bleaching effect without adversely affecting the mineral content or microhardness of dental hard tissues. While the results were promising, the authors acknowledged the need for clinical trials to fully evaluate the safety and efficacy of cold plasma-based bleaching agents in vivo [68] (Figure 5). While these results highlight CAP’s potential as a safer alternative to peroxide-based bleaching agents, the evidence is still limited to in vitro conditions, underscoring the need for well-designed clinical trials.

A 2021 study explored the impact of cold plasma treatment on dental implant surfaces, specifically examining both the efficacy and potential side effects of applying cold plasma to titanium and zirconia implants. The findings demonstrated that cold plasma did not induce any detectable alterations in the chemical composition of the implant materials. Moreover, cold plasma enhanced cell adhesion and proliferation on implant surfaces, suggesting that it can facilitate and accelerate osseointegration, making it a promising approach for improving the biological performance of dental implants [69]. This positions CAP not only as a sterilization method but also as a potential enhancer of implant integration, although long-term in vivo outcomes still require further evaluation.

Beyond its applications in dental medicine, cold plasma has shown significant potential in the field of wound healing and tissue regeneration. It contributes to wound repair by promoting blood coagulation, stimulating cellular responses, and eliminating microorganisms that colonize wound sites [70,71]. These combined effects contribute to an enhanced healing environment.

A 2025 study by Ruidi Gao et al.—the first to examine the effects of cold plasma on wound microbiomes—investigated its impact on both acute and chronic wounds. The authors found that treatment reduced microbial diversity after application. While the results are promising, the study was limited by a small patient sample, and the molecular mechanisms underlying cold plasma–enhanced wound healing remain unclear [70]. Together, these studies demonstrate CAP’s multifaceted role in wound management by combining antimicrobial activity with the promotion of tissue regeneration, yet current evidence is constrained by small patient numbers and short follow-up durations.

In another clinical study conducted in 2021, Jan-Oluf Jensen et al. evaluated the effectiveness of cold plasma in treating chronic wounds. Their results indicated that repeated applications of cold plasma were significantly more effective than single treatments. Multiple exposures not only improved oxygenation within the wounded tissue but also enhanced capillary blood flow. Both of which are critical factors for successful wound healing [71].

Table 3 provides a summary of representative CAP studies in the medical and healthcare domains, highlighting microbial targets, study types, outcomes, and limitations. Overall, medical applications of CAP particularly in wound healing and implant sterilization are progressing toward clinical adoption, whereas areas such as dental bleaching remain at an earlier experimental stage.

### 4.2. Food and Agricultural Applications

Beyond its well-established medical applications, cold plasma has emerged as a versatile tool in several non-medical fields, particularly in agriculture and the food industry. In agriculture, cold plasma has been utilized to enhance seed germination, stimulate plant growth, and aid in soil decontamination by reducing microbial loads. Additionally, it serves as a post-harvest treatment to eliminate pathogens from crop surfaces, contributing to safer and higher-quality produce [72,73].

In the food industry, cold plasma plays a pivotal role in food preservation. Its strong antimicrobial activity enables it to reduce microbial contamination, thereby extending the shelf life of various food products. Moreover, cold plasma has been shown to degrade mycotoxins, inactivate undesirable enzymes, lower pesticide and allergen residues, and preserve bioactive compounds. It also enhances antioxidant activity in treated foods, offering both safety and nutritional benefits [72,73].

Numerous studies have investigated the applications of cold plasma in food safety. For example, a 2021 study evaluated the use of cold plasma for raw milk sterilization. Traditional thermal pasteurization often compromises milk’s sensory qualities and leads to the loss of heat-sensitive vitamins such as vitamin C and B1. The researchers found that cold plasma could significantly reduce microbial counts in milk with 1.5% fat content, more effectively than in milk containing 3% fat, suggesting a fat-dependent efficiency [74]. This indicates that CAP can provide an alternative to thermal pasteurization, offering microbial safety while potentially preserving heat-sensitive nutrients, although variability in efficiency across different fat contents highlights the need for matrix-specific optimization.

Another study in 2022 assessed the efficacy of cold plasma in decontaminating Adobera cheese, a traditional Mexican dairy product. The authors deliberately inoculated 0.5 g samples of cheese with *E. coli* ATCC 25922, *Salmonella* ATCC 13076, and *S. aureus* ATCC 6538 at concentrations of 10^8^ CFU/mL. Following cold plasma treatment, they observed substantial microbial reduction. However, the process also led to oxidation of lipids, which adversely affected the flavor and aroma of the cheese. After 3 min of exposure, 82% of proteins and 99% of free casein were oxidized. The study did not investigate protein hydrolysis or structural changes, which may influence the nutritional and textural properties of the cheese—highlighting a key limitation. The authors recommended that future investigations should evaluate not only microbial inactivation, but also sensory attributes and nutritional impacts of plasma-treated dairy products [75]. These findings illustrate both the strengths and challenges of CAP in dairy preservation: while microbial reduction is substantial, undesirable lipid and protein oxidation can compromise flavor, aroma, and possibly nutritional quality, underscoring the need for integrated sensory and nutritional assessments.

Cold plasma has also proven effective in decontaminating fresh-cut products, such as strawberries and melon slices. It significantly reduces surface contamination by pathogens like *Salmonella* spp. and *E. coli*, which are commonly associated with foodborne illnesses during storage. In addition, cold plasma has been used to prevent spoilage in apples and bananas, effectively prolonging shelf life by suppressing microbial growth [76] (Figure 5).

CAP shows strong potential for improving food safety by reducing microbial loads without thermal damage, yet its translation to industrial practice remains limited by concerns about sensory alterations, nutrient stability, and the lack of standardized treatment protocols.

### 4.3. Water and Environmental Applications

Cold plasma has recently emerged as a promising alternative to traditional wastewater treatment technologies. Its water purification capabilities arise from the synergistic effects of UV radiation, ozone generation, reactive species formation, and intense electric fields [77,78]. Unlike conventional methods that often rely on chemicals or thermal processes, cold plasma offers a chemical-free and energy-efficient approach to water treatment.

Several studies have demonstrated cold plasma’s bactericidal efficiency. For instance, Zohreh Rashmei et al. reported that cold plasma effectively inactivated high concentrations of *Enterococcus faecalis* and *E. coli*, confirming its potential in microbial disinfection of contaminated water [79].

In addition to its antibacterial activity, cold plasma exhibits potent antiviral properties, primarily due to the generation of ROS and RNS. These species can degrade viral envelopes and inhibit replication, thereby effectively inactivating viruses such as norovirus, adenovirus, hepatitis A virus, HSV-1, and bacteriophages like MS2, T4, and φX174 [80].

Cold plasma has also been explored for its ability to degrade organic contaminants, including azo dyes, and reduce heavy metal concentrations in water—specifically Pb, Cd, Fe, and Mn. However, studies conducted by Dung Van Nguyen et al. showed that while cold plasma is effective against many metal pollutants, it has limited efficacy in removing arsenic residues. Furthermore, they observed that plasma treatment may result in the formation of nitrate species during water purification, which could present an additional consideration for long-term application [78,79] (Figure 5).

Another important environmental application of cold plasma is in the removal of pesticides from water sources. It has been shown to break down these toxic compounds without generating hazardous byproducts, highlighting its environmental safety [81].

Cold plasma has also shown strong potential in eliminating pharmaceutical residues from wastewater. In a study by Aleksandra Wypart-Pawłul et al., the removal efficiency of several drugs was evaluated, including diclofenac (DFC), sulfamethoxazole (SMX), trimethoprim (TMP), carbamazepine (CBZ), and caffeine (CAF). The authors found that cold plasma treatment resulted in a significant reduction in most compounds, with TMP reduced by 98%, SMX by 73%, and DFC by 91%. However, the method was ineffective for caffeine, and only 1% of CBZ was removed, indicating compound-specific variability in degradation efficiency [82].

These results align with earlier findings by Ema Wielogorska et al., who demonstrated that cold plasma efficiently degrades antibiotics in wastewater without promoting bacterial resistance. Their study highlighted that removal efficiency depends on factors such as pH, the presence of polar macromolecules, and the initial contaminant concentration. The authors recommended further research to optimize treatment conditions and develop predictive models for cold plasma performance across different wastewater compositions [83,84].

Cold plasma has shown considerable promise in degrading pharmaceutical residues in wastewater, with high efficiency against some antibiotics but limited activity against more recalcitrant compounds, such as caffeine and carbamazepine. This variability highlights the need to better understand compound-specific degradation mechanisms and optimize treatment parameters. Although the lack of induced resistance is an advantage, further research is needed to improve consistency and assess scalability before CAP can be applied to large-scale wastewater treatment.

## 5. Cold Plasma in Combination with Other Antimicrobial Strategies

CAP has become known as a viable non-thermal method for microbial decontamination across different applications, including food processing and medical therapies. Its capability to produce reactive oxygen and nitrogen species under ambient settings renders it an efficient instrument against several diseases [85]. Researchers are actively investigating the integration of CAP with alternative antibacterial methods to augment its efficacy and address potential limitations. One solution utilizes plasma-activated water (PAW), which has demonstrated considerable potential in food sector applications. PAW is applicable for microbiological decontamination of food products, reduction in pesticide residues, and disinfection of materials that contact food [86]. The integration of CAP with additional technologies has been examined to enhance its efficacy. Research has investigated the synergistic effects of CAP and antibiotics in the treatment of methicillin-resistant *S. aureus* (MRSA) biofilms, revealing superior inactivation compared to singular therapies [87]. Recent studies have concentrated on integrating CAP with additional light-based antimicrobial methods, including antimicrobial blue light, photodynamic inactivation, pulsed light, and UV light. These integrated techniques seek to use the multitarget efficacy of many modalities, potentially mitigating the danger of microbial tolerance emergence [88]. The combined effects of airborne acoustic ultrasound and plasma-activated water have demonstrated potential in improving antimicrobial effectiveness against bacterial biofilms, suggesting possible applications in the food and biomedical sectors [89] (Figure 5).

### 5.1. Synergistic Effects with Antibiotics

The integration of CAP with antibiotics has demonstrated encouraging outcomes in addressing antibiotic-resistant bacterial strains, presenting a potential remedy for the escalating challenge of multidrug-resistant (MDR) microorganisms. CAP, consisting of several bactericidal species, demonstrates remarkable bactericidal efficacy against microorganisms via intricate and varied pathways [90,91,92]. CAP has demonstrated encouraging outcomes in clinical environments, presenting prospective benefits compared to conventional antibiotic therapies for specific conditions: CAP has proven efficient against antibiotic-resistant bacteria, rendering it a preferable option to conventional antibiotics in certain instances [49]. When administered alongside antibiotics, CAP can augment their effectiveness against resistant microorganisms. Plasma-activated saline (PAS), a derivative of CAP, has demonstrated the ability to enhance the efficacy of medicines against MRSA biofilms. The concurrent administration of PAS with rifampicin and vancomycin diminished roughly 6.0-log10 MRSA cells in biofilms and significantly mitigated systemic infection in a mouse model [86]. The amalgamation of CAP with natural extracts has exhibited synergistic effects against multidrug-resistant microorganisms. The amalgamation of *Cichorium intybus L*. (Chicory) extract with argon plasma treatment markedly diminished the metabolic activity of *P. aeruginosa* and *E. coli* biofilms, demonstrating considerable membrane disruption [89]. This indicates that the amalgamation of CAP with additional antimicrobial drugs may augment its efficacy against resistant bacteria. In conclusion, the integration of cold plasma and antibiotics is a viable strategy to address antibiotic-resistant bacteria. This synergistic action may enhance treatment outcomes, diminish the development of antibiotic resistance, and perhaps offer novel techniques for tackling the escalating issue of multidrug-resistant bacterial infections [90,91].

Although CAP exhibits remarkable bactericidal efficacy at suitable dosages, certain bacteria may acquire tolerance mechanisms during therapy [51]. Conversely, antibiotics generally focus on particular biological mechanisms, such as cell wall construction or protein synthesis, potentially resulting in the emergence of resistance over time. Combining CAP with antibiotics can augment their effectiveness by promoting antibiotic accumulation within bacteria [92]. CAP presents a viable alternative to conventional antibiotics owing to its diverse bactericidal processes and capacity to address antibiotic-resistant microorganisms. The synergistic benefits of integrating CAP with antibiotics or other antimicrobial agents provide an innovative approach to combat the escalating challenge of antimicrobial resistance [93].

CAP demonstrates potential, although its effectiveness may fluctuate based on the particular application and the pathogens implicated. In the treatment of burn wounds infected with *P. aeruginosa,* CAP proved to be less effective than standard antibacterial wound irrigation solutions [94]. Moreover, specific bacterial strains, such as particular *S. aureus*, may demonstrate tolerance to CAP therapy [95]. Although CAP shows promise as an alternative or supplementary treatment to conventional antibiotics, its efficacy may differ depending on the particular illness and bacteria involved. Additional investigation is required to enhance CAP treatment methods and comprehensively comprehend its long-term effects in comparison to conventional antibiotics [49]. The incorporation of CAP as an effective treatment for complex wounds is substantiated by increasing clinical safety and efficacy evidence [96]; nonetheless, further extensive clinical trials are necessary to define its role in diverse medical applications.

Triple antibiotic paste, comprising ciprofloxacin, metronidazole, and minocycline, is employed to eliminate biofilms during root canal disinfection procedures. The efficacy against *E. faecalis* has been established, particularly when incorporated into a scaffold that improves antibiotic retention and administration, resulting in a substantial decrease in biofilm formation and bacterial viability [97].

CAP has emerged as an innovative method for effectively attacking biofilms, owing to its capacity to produce reactive species without inflicting heat harm on adjacent tissues. While there is a scarcity of particular studies examining CAP’s effects on *E. faecalis* in root canals, CAP’s overarching mode of action is the disruption of bacterial cell walls and the degradation of biofilms. The triple antibiotic paste exhibited the most pronounced antibacterial efficacy, with a mean log CFU of 0, signifying total bacterial eradication. Conversely, the control group exhibited a mean log CFU of 8.87 ± 0.21. The plasma group exhibited a significant decrease in bacterial count, with a mean log CFU of 8.09 ± 0.18, in contrast to the control group, which presented a greater bacterial load of 9.15 ± 0.26. The results indicate that although both treatments were successful, the triple antibiotic paste demonstrated greater antibacterial activity [98].

### 5.2. Enhancement of Bacteriophage Therapy

CAP has demonstrated encouraging outcomes in augmenting bacteriophage therapy via synergistic effects. CAP produces reactive oxygen and nitrogen species (ROS/RNS) that can harm bacterial cell membranes and enhance their vulnerability to phage infection [99]. This combinatorial method has shown enhanced efficacy in addressing multidrug-resistant bacterial infections relative to monotherapy. The synergistic interaction between CAP and bacteriophages is notably intriguing regarding biofilm elimination [100]. This synergy likely results from CAP’s capacity to disrupt the extracellular matrix of biofilms, facilitating enhanced penetration of phages to access their bacterial targets. The addition of antibiotics can further augment the synergistic impact between CAP and phages. Research indicates that the integration of phages with cell wall-targeting antibiotics and CAP treatment enhances bacterial eradication and biofilm elimination [100]. This tripartite method may provide an effective technique for addressing intricate, antibiotic-resistant illnesses, especially in instances where conventional medicines have been ineffective.

CAP treatment may induce cellular membrane damage mostly by lipid peroxidation due to the production of ROS [101]. The disruption to the membrane may render bacterial cells more vulnerable to phage infection, however this is not expressly articulated in the supplied literature.

Furthermore, CAP has demonstrated the ability to alter the immunoreactivity of human polymorphonuclear cells (PMNs), augmenting the expression of integrins and selectins on their surfaces [102]. Although not directly linked to phage efficacy, research indicates that CAP can influence immunological responses, perhaps impacting bacterial susceptibility to phages indirectly.

Phage treatment is usually considered safe, with significant adverse events being exceedingly rare [99]. Numerous investigations, including animal research, clinical case reports, and clinical trials, have indicated negligible safety issues related to phage therapy [103]. Phage therapy seems to be predominantly safe; nevertheless, further research is necessary to comprehensively ascertain its safety profile, particularly in conjunction with other treatments such as CAP. Standardized safety measures and reporting methodologies are essential for forthcoming research [104]. The establishment of national frameworks for the magistral preparation of tailored phage products may alleviate safety issues while offering prompt remedies for challenging illnesses [105].

### 5.3. Cold Plasma and Nanotechnology-Based Antibacterial Agents

Cold plasma and nanotechnology-derived antibacterial agents have surfaced as viable alternatives to conventional antibiotics in the fight against bacterial infections and the escalating issue of antimicrobial resistance. Cold plasma exhibits notable antibacterial effects via multiple pathways [106]. When integrated with nanotechnology, these methods provide improved effectiveness and precise delivery of antimicrobial drugs. Metal-based nanoparticles exhibit exceptional antibacterial efficacy owing to their distinctive physicochemical characteristics. Silver nanoparticles (AgNPs) demonstrate significant antibacterial properties against various bacteria, with their effectiveness mostly influenced by size, shape, and chemical composition [107].

Numerous instances of nanotechnology-derived antibacterial compounds integrated with cold plasma encompass silver nanoparticles, zinc oxide nanoparticles, and copper nanoparticles. These nanoparticles exhibit synergistic effects when combined with cold plasma treatment. Silver nanoparticles in conjunction with cold plasma have exhibited increased antibacterial efficacy against *E. coli* and *S. aureus* [108,109].

The integration of cold plasma and nanotechnology has demonstrated potential in tackling the escalating issue of antibiotic resistance. Gold nanoparticles infused with cefotaxime (a third-generation cephalosporin antibiotic) and subjected to cold plasma exhibit enhanced antibacterial efficacy against multidrug-resistant strains of *E. coli*, *Klebsiella oxytoca*, and *P. aeruginosa* in comparison to the antibiotic alone [110]. The antibacterial actions of metal-based nanomaterials encompass energy conversion and electron transfer activities, resulting in the breakdown of bacterial cell membranes and metabolic pathways [111]. Furthermore, the integration of cold plasma and nanoparticles facilitates the development of intelligent, stimuli-responsive antibacterial agents that can be activated by external stimuli such as light or ultrasound [52].

### 5.4. Use in Combination with UV or Chemical Disinfectants

When integrated with other disinfection methods like UV or chemical treatments, CAP can provide improved antimicrobial effectiveness. The integration of CAP with UV irradiation constitutes an effective method for microbial inactivation. Ultraviolet radiation, namely at wavelengths between 220 and 260 nm, has demonstrated significant efficacy against viruses such as SARS-CoV-2 variants [112]. The combination of CAP, which produces reactive species and physical variables such as UV light, may result in enhanced disinfection efficacy [112,113]. The use of CAP with chemical disinfectants such as H_2_O_2_ has demonstrated potential in augmenting antibacterial efficacy. H_2_O_2_, a potent oxidizing agent, exhibits extensive efficacy against bacteria, fungi, and viruses [114]. The amalgamation of CAP and H_2_O_2_ may enhance the oxidative damage induced by both techniques, resulting in more efficient microbial inactivation. The application of cold plasma alongside UV or chemical disinfectants provides a multi-faceted strategy for bacterial inactivation. This strategy corresponds with the increasing demand for eco-friendly and efficient disinfection techniques [114,115]. Nonetheless, it is crucial to acknowledge that although these integrated methodologies exhibit potential, additional study is required to refine their implementation and evaluate any possible effects on the treated materials or surfaces [87,116].

The synergistic approach of combining cold plasma and nanotechnology offers a promising avenue for developing effective antibacterial agents. These innovative strategies have the potential to overcome the limitations of conventional antibiotics and provide new solutions for combating bacterial infections and antimicrobial resistance [117,118]. However, further research is needed to fully understand the long-term effects and potential risks associated with these novel antibacterial approaches [106,119]

### 5.5. Critical Comparison of Combination Strategies

Among the investigated combinations, the integration of CAP with antibiotics is the most advanced, supported by in vitro, in vivo, and even clinical evidence. For instance, plasma-activated saline enhanced the efficacy of rifampicin and vancomycin against MRSA biofilms and reduced systemic infection in a mouse model [86]. CAP has also shown promise in clinical applications such as wound treatment, with increasing safety and efficacy evidence reported [96]. These findings indicate that CAP–antibiotic strategies are relatively mature compared to other combinations. By contrast, other synergistic strategies remain more exploratory. The combination of CAP with bacteriophages has shown potential in biofilm elimination and MDR infections [99,100], but research is still at an early stage, with safety and immunological effects requiring further clarification. Similarly, CAP combined with nanotechnology-derived antibacterial agents, such as silver, zinc oxide, and copper nanoparticles, has demonstrated enhanced antimicrobial activity [108,109,110], yet these studies remain primarily laboratory-based. Questions regarding long-term effects, potential toxicity, and applicability beyond proof-of-concept still need to be addressed. Likewise, the integration of CAP with UV or chemical disinfectants has shown synergistic effects in microbial inactivation [112,114], but practical deployment and material safety require further study. To better illustrate the progress and limitations of different synergistic approaches, a comparative summary of CAP combination strategies is provided in Table 4. Overall, CAP–antibiotic combinations are the most advanced and closest to translation into practice, particularly for biofilm control and resistant infections. In contrast, CAP combined with bacteriophages, nanotechnology, or chemical/UV disinfectants remains largely exploratory, highlighting areas where additional investigation is necessary before clinical or industrial adoption.

There are encouraging prospects for increased antimicrobial efficacy when CAP is combined with several antibacterial modalities. But it also brings with it serious problems with cytotoxicity, safety, and regulatory barriers. Here is a more thorough explanation of these points:

1. CAP with Antibiotics: Because of the increased reactive species produced, there is a higher chance of tissue injury when CAP and antibiotics are used together. CAP can decrease the bacterial load and increase the effectiveness of antibiotics, but its use must be carefully managed to avoid cytotoxicity in nearby healthy tissues [124]
2. CAP with Bacteriophages: Bacteriophages are known to eliminate unwanted effects on human cells due to their specificity to bacteria. However, due to its strong bactericidal action and the generation of reactive species, the synergistic usage of CAP may still present an indirect cytotoxicity risk. Phage resistance and the immunological response to phage therapy are additional issues that require more research [125,126].
3. CAP with Antibacterial Agents Based on Nanotechnology: Although nanoparticles provide improved penetration and targeted distribution, their size, shape, and surface chemistry can all increase their risk of cytotoxicity. For instance, because silver nanoparticles can interact with biological components and produce ionic forms, they can cause cytotoxicity and genotoxicity [127]. When combined with CAP, a thorough assessment of the behavior of the nanoparticles under conditions of oxidative stress generated by the plasma is necessary [128].
4. CAP using Chemical or UV Disinfectants: Although these combinations provide better disinfection, there is a chance that the materials will deteriorate and leave behind residues that could harm the environment and human health. Strict safety precautions are required because the oxidative effects of CAP combined with UV or chemical agents can increase these dangers [124].

Despite its potential to combat bacteria that are resistant to drugs, bacteriophage therapy currently faces numerous regulatory obstacles because of its lack of standardization and solid clinical efficacy data [129]. Given the possibility of phage mutation and the emergence of resistance, regulatory agencies are wary. While integrating nanotechnology offers promise, current regulations lack specificity for nanoparticles and CAP. Thorough assessments of long-term effects are essential, and comprehensive evaluation standards are needed to understand their behavior in biological systems and ensure safe application [125,128]. Intricacy of formulations, meeting current regulatory criteria is made more challenging by the intricacy of mixing CAP with numerous medicines. Because each element may interact differently, new assessment techniques that take into account the cumulative impacts are necessary [130]

## 6. Conclusions

Cold plasma represents a promising non-thermal antimicrobial approach with the ability to inactivate a wide spectrum of pathogenic bacteria, including multidrug-resistant strains. Its unique mechanisms generation of reactive oxygen and nitrogen species, disruption of bacterial membranes, oxidative damage to intracellular components, and modification of nucleic acids and proteins differentiate it fundamentally from conventional antibiotics. The efficiency of cold plasma is influenced by plasma parameters, bacterial species, and environmental conditions, which highlights the need for careful optimization. Despite its potential, several challenges remain before widespread clinical and industrial adoption can be achieved. These include the risk of cytotoxicity to host tissues, lack of standardized plasma devices, variability in treatment protocols, and cost considerations. Addressing these limitations will be essential to ensure safe and reproducible outcomes. Looking forward, cold plasma holds promise not only as a standalone intervention but also as a complementary strategy alongside existing antimicrobials, which may help mitigate antibiotic resistance. Future work should focus on translational studies, regulatory frameworks, and comparative evaluations with current technologies to accelerate its pathway toward practical application.

## 7. Future Prospects

Despite its proven effectiveness, further research is needed to fully understand and optimize cold plasma treatments for clinical, food, and environmental applications. Future work should focus on the following:

1. Standardization of treatment protocols across different plasma sources, treatment parameters, and application fields to ensure reproducibility, comparability between studies, and safety benchmarks.
2. Mechanistic studies at the molecular level, particularly regarding the interaction of reactive species with bacterial biomolecules, biofilm matrix components, and potential resistance pathways, to better predict efficacy and limitations.
3. Development of portable, cost-effective, and scalable plasma devices for deployment in hospitals, food-processing facilities, agricultural settings, and remote or resource-limited environments, addressing current scalability challenges.
4. Assessment of long-term safety and cytotoxicity, especially in applications involving human tissues, medical devices, and food products, with particular emphasis on possible impacts on sensory qualities in food and host microbiota balance in clinical use.
5. Clinical trials with larger patient cohorts, as current studies are limited by small sample sizes and short follow-up periods, which restrict generalizability and regulatory approval.
6. Regulatory considerations and approval frameworks: collaborative efforts with health and food safety authorities (e.g., FDA, EFSA, Codex) are necessary to establish guidelines for plasma-based technologies to transition from experimental to mainstream adoption.
7. Optimization of plasma treatment in food and water applications, addressing current gaps such as variability in microbial inactivation across different matrices and potential effects on nutritional and organoleptic properties of foods.
8. Integration with complementary technologies (e.g., antimicrobials, coatings, nanomaterials) to enhance efficacy and reduce variability in outcomes across clinical, industrial, and environmental contexts.

## Author Contributions

Conceptualization, Y.A.-G.M., N.E.E., F.M.E., Y.A. and S.S.; software, G.Y., K.M. and F.M.E.; formal analysis, Y.A.-G.M., N.E.E., F.M.E., Y.A. and S.S.; writing—original draft preparation, Y.A.-G.M., N.E.E., F.M.E., Y.A. and S.S.; writing—review and editing, Y.A.-G.M., N.E.E., F.M.E., Y.A., S.S., K.M. and G.Y.; supervision, Y.A.-G.M. and S.S. funding acquisition, K.M. All authors have read and agreed to the published version of the manuscript.

## Figures and Tables

**Table 1:** Summary of CAP effects on different bacterial species.

Bacterial Species	Exposure Time	Main Observed Effects	Representative Studies
*P. aeruginosa*	Not specified	Oxidation of lipid membrane; disruption of intracellular ion balance; alteration of membrane permeability; morphological deformation; bacterial inactivation	[30]
*E*. *coli* NCTC 12900	1, 3, and 5 min	Reduction: 2 log (1 min + 24 h storage); 3.6 log (3 min direct); 2.3 log (3 min indirect); 6 log (5 min direct); 8 log (5 min indirect). Cell wall disrupted by ROS; cleavage of C–C, C–O, and C–N bonds in LPS and peptidoglycan; lipid peroxidation; collapse of cell envelope	[33]
*S. aureus* ATCC 25923	1, 3, and 5 min	Complete elimination after 1–5 min with 24 h storage (direct or indirect); complete elimination after 3–5 min + 1 h storage. Without storage: 1 min = 1.8 log reduction, 5 min = 6.1 log reduction. ROS penetrated cell wall, accumulated inside, internal components damaged; higher intracellular ROS than *E. coli*	[33]
*Bacillus cereus*	Not specified	Breakdown of cell membrane; ROS penetrated cell and attacked vital macromolecules; damage to cellular homeostasis; cell death	[31]
*E*. *coli* CIP 53126	10 min	Pore formation inside cell membrane; complete membrane disruption; morphological alterations; bacterial inactivation	[36]

**Table 2:** Comparative Summary of Reactive Species, Flow Rate Effects, and Bacterial Responses.

Reactive Species	Rate Flow Effect	Bacterial Response	References
Reactive oxygen species	Target protein, lipid, DNA.	Severe damage to bacterial cells.	[55,60]
Free radicals	Breaking peptide bonds and unfolding of proteins in bacterial cell wall.	Change in protein three dimensions.	[56,60]
Reactive oxygen species	Excessive oxidative stress.	Programed cell death and necrosis.	[60]
Singlet oxygen	React with internal component.	Cell damaging.	[59]

**Table 3:** Summary of CAP Studies.

Domain	Microbial Target	Study Type	Outcomes	Limitations	Representative Study
Sterilization of surgical implants	*Pasteurella multocida*	In vivo [on rabbits]	Complete inactivation of bacteria without any visible tissue damage or erythema in the epidermis, dermis, subcutis, or muscle layers	Not tested on other bacterial species	[68]
Environmental decontamination [hospitals]	*S. aureus* and MRSA biofilms	In vitro [lab experiment]	Complete disruption of MRSA biofilms	Conducted in lab, not in real hospital environments	[10]
Dentistry [bleaching agent]	NA	In vitro	Treated water with cold plasma exhibited strong bleaching effect without affecting mineral content or microhardness of dental hard tissues	In vitro only, no in vivo evaluation of safety and efficiency	[69]
Dentistry [Dental implant treatment]	NA	In vitro	Cold plasma did not affect chemical composition of implant materials and enhanced cell adhesion and proliferation on implant surfaces	No in vivo testing	[69]
Chronic wound treatment	Not specified	Clinical study	Multiple exposures of cold plasma improved oxygenation of tissues and enhanced capillary blood flow in tissues	No mention of long-term effects of cold plasma use in wound treatment	[71]

**Table 4:** Synergistic strategies combining CAP with other antimicrobial approaches.

Combination Strategy	Partner Modality	Target Organisms [Species]	Study Type	Outcomes	Limitations	References
CAP with Antibiotics	Antibiotics	*Listeria monocytogenes*, *Escherichia coli*	In vitro/In vivo	Enhanced antibacterial efficacy, synergy against bacteria	Potential for tissue damage, limited human studies	[120]
CAP with Nanotechnology-based Agents	Nanoparticles	Mixed bacterial biofilms	In vitro/In vivo	Increased biofilm disruption, targeted antimicrobial delivery	Nanoparticle toxicity, regulatory challenges	[121,122]
CAP with UV or Chemical Disinfectants	UV/Chemical disinfectants	Broad spectrum, environmental surface pathogens	In vitro	Enhanced decontamination, reduced pathogen numbers	Potential material degradation, environmental impact	[120,122]
CAP and Bacteriophage Therapy	Bacteriophages	Bacterial pathogens	In vitro/In vivo	Synergistic bactericidal effects, improved phage function	Specificity of phages, potential genetic adaptation by targets	[120,123]

## Data Availability

All data included in this review are available with the corresponding author on reasonable request.
